# Identification of ubiquitin/ubiquitin-like protein modification from tandem mass spectra with various PTMs

**DOI:** 10.1186/1471-2105-12-S14-S8

**Published:** 2011-12-14

**Authors:** Chiyong Kang, Gwan-Su Yi

**Affiliations:** 1 Department of Bio and Brain Engineering, KAIST, Daejeon 305-701, South Korea

## Abstract

**Background:**

Various solutions have been introduced for the identification of post-translational modification (PTM) from tandem mass spectrometry (MS/MS) in proteomics field but the identification of peptide modifiers, such as Ubiquitin (Ub) and ubiquitin-like proteins (Ubls), is still a challenge. The fragmentation of peptide modifier produce complex shifted ion mass patterns in combination with other PTMs, which makes it difficult to identify and locate the PTMs on a protein sequence. Currently, most PTM identification methods do not consider the complex fragmentation of peptide modifier or deals it separately from the other PTMs.

**Results:**

We developed an advanced PTM identification method that inspects possible ion patterns of the most known peptide modifiers as well as other known biological and chemical PTMs to make more comprehensive and accurate conclusion. The proposed method searches all detectable mass differences of measured peaks from their theoretical values and the mass differences within mass tolerance range are grouped as mass shift classes. The most possible locations of multiple PTMs including peptide modifiers can be determined by evaluating all possible scenarios generated by the combination of the qualified mass shift classes.The proposed method showed excellent performance in the test with simulated spectra having various PTMs including peptide modifiers and in the comparison with recently developed methods such as QuickMod and SUMmOn. In the analysis of HUPO Brain Proteome Project (BPP) datasets, the proposed method could find the ubiquitin modification sites that were not identified by other conventional methods.

**Conclusions:**

This work presents a novel method for identifying bothpeptide modifiers that generate complex fragmentation patternsand PTMs that are not fragmented during fragmentation processfrom tandem mass spectra.

## Background

Identification techniques of post-translational modification (PTM) from mass spectra were developed to understand the functions of PTMs in biological pathways. Ub and Ubls play important roles in regulatory networks in most of cellular processes and the importance of identification of Ub and Ubls is increased.

From tandem mass spectra, it is difficult to identify peptide with considering all possible PTMs with high performance. Standard database search algorithms (Mascot [1], SEQUEST [2,3], X!Tandem [4]) are well-known tools for peptide identification and these database search programs can identify restricted number of modifications. PTM identification methods which can support unrestricted PTM identification are introduced to cover various PTMs. To reduce computational complexity, candidate peptides are filtered with external tools like standard database search algorithms based on peptide fragment fingerprinting (PFF) or sequence tag extraction based programs [5]. Peptide filtering supports PTM identification tools considering various possibilities of PTMs in proper time by reducing target peptide DB or direct peptide identification. From identified peptide by SEQUEST, P-mod [6] calculates shifted mass from precursor ion mass and detects the sequence location of PTM. PTM-Explorer [7] detects PTMs with identified peptide sequence information from standard database search algorithms. Recently, QuickMod [8] that searches PTM using prebuilt library spectra showed favorable performance against other PTM analysis methods.

**Figure 1:**
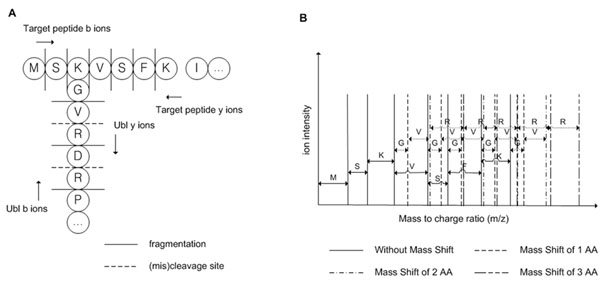
**Various mass shifts that could be generated from a Ubl.** (a) An example of theoretical fragmentation patterns and cleavage sites from UFM1_HUMAN. (b) A theoretical spectrum of b-ions from Ubl-modified target peptide, without y-ions from Ubl-modified target peptide and b-ions from Ubl.

Relatively simple chemical modifications including acetylation, methylation, and phosphorylation are successfully identified with prior PTM identification tools. However, more specified method is required to identify peptide modifiers including Ub and Ubls. Various mass shifts could be generated from peptide modifiers while only one mass shift is generated from usual PTMs, because peptide modifiers could be digested and fragmented in the MS/MS analysis. Fig. 1 shows various mass shifts that could be generated from a peptide modifier. Not only target peptides but also peptide modifiers are cleaved and fragmented hence generated complex spectrum from various mass shifts (Fig. 1.A). Miscleavage of peptide modifiers can provoke more peaks by adding mass shifts of miscleaved peptide modifier amino acids. As y-ions from peptide modifier are attached to target peptide, theoretical spectrum of Ub/Ubl-modified peptide is consisted by b-ions from Ubl-modified target peptide (Fig. 1.B), y-ions from Ubl-modified target peptide, and b-ions from Ubl. For specified identification method for peptide modifier, SUMmOn [9] suggested a new method with automatic pattern recognition by matching diagnostic SUMO fragment ion series in tandem mass spectra. SUMmOn is specified PTM identifier especially for SUMOylation, and it suggests a method to identify peptide modifiers that produce fragment ion series. From measured spectra, SUMmOn considers intense peaks per 100-Da window and may ignore peaks with low intensity which may contain peaks from fragment ion series, which are not noise peaks. SUMmOn requires precursor ion mass of the target peptide while there could be absence of efficient conjugate fragmentation [10] and part of spectra in the tandem mass spectra datasets have lack of information about precursor ion mass.

We present a new method that can identify not only peptide modifiers such as Ub and Ubls but also various PTMs from tandem mass spectra. Suggested method detects mass shift classes and identifies PTMs with considering all known peptide modifiers. All theoretical mass shifts from peptide modifiers are matched with mass shift classes from MS/MS spectra without peak filtering. The proposed method can attempt to identify peptide modifiers though there is lack of information of precursor ion mass. All known PTMs and unknown mass shifts can be considered together while peptide modifiers identification.

## Methods

### Unrestricted PTM identification

**Figure 2:**
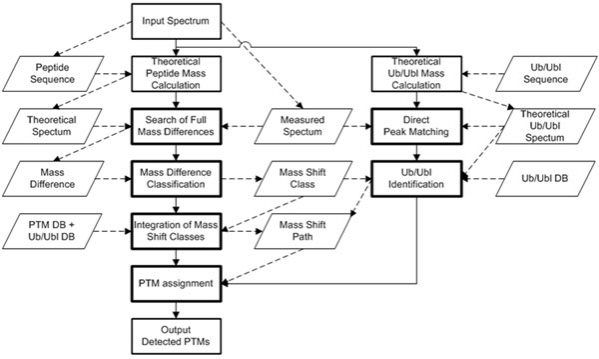
The flow chart of the proposed algorithm.

**Figure 3:**
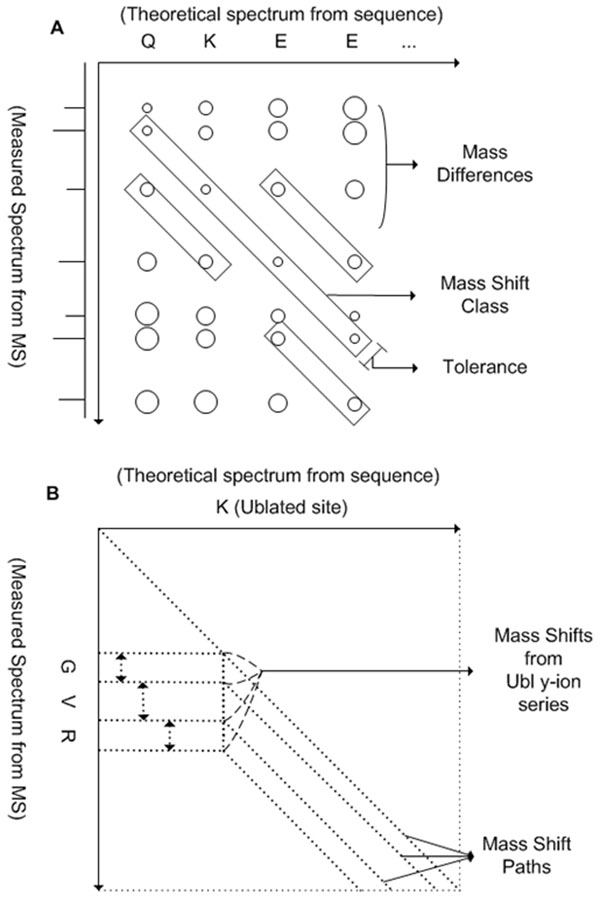
**The process of mass shift path finding.** (a) Equivalent mass differences are clustered as mass shift classes and evaluated. (b) Qualified mass shift classes are connected into mass shift sets and evaluated to find the mass shift paths. Fragmented Ubl produces various mass shifts while most of non-peptide PTMs produce only one mass shift.

Unrestricted PTM identification method based on our prior algorithm [11] is mainly applied for detecting PTMs that are not fragmented during fragmentation process such as CID. Our prior algorithm can consider all possible PTM candidates without restriction on the precursor mass tolerance and hence can also be applied to identification of peptide modifiers with a large mass by detecting various mass shift classes generated from fragment ions of peptide modifiers. Input tandem mass spectra have information of spectra with identified peptide sequences by standard database search algorithms. The theoretical fragment ion masses are calculated from the peptide sequence. With four core stages for PTM identification from our prior algorithm (a search of full mass differences, mass difference classification, integration of mass shift classes, and PTM assignment), two stages are added to identify Ub and Ubls: direct peak matching of Ub/Ubl b-ions with measured peaks and Ub/Ubls identification by matching of Ub/Ubl y-ions with mass shift classes. (Fig. 2) Fig. 3.A demonstrates mass difference classification. Mass differences between all measured mass peaks without peak filtering and theoretical mass of fragment ions are calculated and clustered as mass shift classes with mass tolerance range. To filter computational artifact mass shift classes, mass shift classes are evaluated based on the intensity and deviation of mass peaks, and on the number of mass differences in the class. Multiple PTMs can be identified by evaluating correlations between measured spectrum and theoretical spectra from mass shift sets that indicate all possible scenarios from combination of qualified mass shift classes.

### Identification of Ub/Ubls

**Figure 4:**
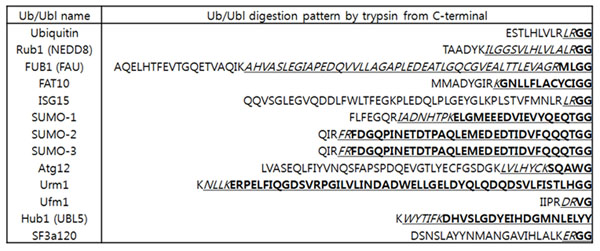
**The Human Ub/Ubl sequences with digestion pattern by trypsin.** Bold: no miscleavage, italic or bold with underline: 1 miscleavage, full polypeptide sequences: 2 miscleavages.

PTM identification tools usually try to identify PTM by comparing detected mass shift with PTM database such as Unimod [12]. Unimod has the information of protein modifications for mass spectrometry data including Ub and SUMOylations but not fully covers all Ubls. To cover all Ubl, all known Ubls and putative Ubls are computationally digested considering 0~2 miscleavages and the mass of the fragmented peptides are calculated for identification of Ubls. Not only for identifying miscleaved Ub/Ubls, to analyze spectrum with lack of precursor ion mass information of the target peptide due to absence of efficient conjugate fragmentation, miscleavage should be considered together. Total 13 Ub/Ubl sequences are selected for mass shifts calculation of human Ub/Ubls: Ub, NEDD8 (Rub1), FUBI (FAU), FAT10, ISG15, SUMO-1, SUMO-2, SUMO-3, Atg8, Atg12, Urm1, UFM1, and SF3a120. (Fig. 4) With calculated mass shifts of Ub/Ubls digested by trypsin, we may try to identify Ub/Ubls with prior standard database search algorithms or PTM identification programs. However, as Fig. 1.A described, peptides are not only digested by enzymes but also fragmented by dissociation instruments such as CAD or ECD in tandem mass spectra. Most of PTM identification algorithms usually try to find mass shift of GlyGly (diglycine modification) or LeuArgGlyGly on K residue to identify Ub, without considering fragmented peptides of Ub y-ions such as Gly and ArgGlyGly. Mass shift of diglycine modification (114.1 Da) is being used for identification of Ub, NEDD8 (Rub1), and ISG15. However, iodoacetamide (IA) that usually used cysteine alkylating agent also has similar mass shift to diglycine modification that could cause false positive identification of Ub/Ubls. [10,13] The proposed method considers both digested patterns and fragmented patterns of Ub/Ubls peptides with or without miscleavage. A mass shift can shift all following peaks with target peptide sequence manner. As Ub/Ubls can have more than one mass shift at lysine residue, following peaks after lysine residue from target peptide can be shifted diversely. (Fig. 1.B) For detecting variously fragmented y-ions from Ub/Ubls, our method tries to build multiple mass shift paths from mass shift classes. Fig. 3.B shows various mass shift paths from fragmented Ub/Ubls. Matched mass shift classes with mass shift of theoretical fragmented y-ions from Ub/Ubls are used to build mass shift paths. As sequence tag can be generated from the mass differences of mass peaks, attached Ub/Ubls sequence can be founded from the mass differences of mass shift paths. The proposed method identifies peptide modifiers by matching of Ub/Ubl y-ions with mass shift classes in mass shift paths.While y-ions of Ub/Ubls are attached on target peptide and matched with mass shift classes, b-ions of Ub/Ubls that are not attached to target peptide are mainly used on direct peak matching. Theoretical b-ions from Ub/Ubls spectrum with considering miscleavage are matched with measured spectra. The proposed method can try identification of Ub/Ubls with Ub/Ubl b-ions if there is enough length of fragment b-ion series due to fragmentation pattern or miscleavage. Fig. 4 shows human Ub/Ubl sequences and digestion pattern with trypsin. FAT10, SUMO-1, SUMO-2, SUMO-3, Urm1, and Hub1 are digested with longer than 5 amino acid lengths without miscleavage. In addition, all human Ub/Ubl sequences are digested as no shorter than 4 amino acid lengths with 1 miscleavage which is enough to build sequence tags. From peptide modifier sequences, theoretical fragment ion masses are calculated and b-ions from peptide modifiers are directly matched with measured mass peaks to support the identification of peptide modifiers in the proposed method.

## Results

### Identification of mixed PTMs including Ub/Ubls

Simulated spectra datasets are generated for validation of the proposed method in various conditions. Total 94717 human protein sequences from Uniprot are digested allowing miscleavages for simulated spectra generation. All biological PTMs in Unimod and 13 kinds of Ub/Ubls allowing miscleavage 0 to 2 are randomly attached on digested peptide sequences. Biological PTMs in Unimod are 275 selected PTMs with target site specificity and classification as post-translational, co-translational, pre-translational, multiple, N-linked glycosylation, O-linked glycosylation, and other glycosylation. An addition of 0 to 20% missing peaks and 0 to 50% noise peaks, with tolerances of 0.5 Da was processed with the simulated spectra. Table 1 demonstrates analysis result of simulated spectra datasets. The average accuracy of the proposed method on simulated spectra with randomly attached 1 PTM including biological PTMs in Unimod and Ub/Ubls was 91%. Three kinds of simulated spectra datasets are generated for 2 PTMs analysis. First, 2 PTMs with only biological PTMs are randomly attached to simulated spectra and the average accuracy of the proposed method on this spectra dataset was 53%, which demonstrates a better accuracy than in previous methods [14,15]. Second, 2 PTMs with only Ub/Ubls are randomly attached to simulated spectra and average accuracy of the proposed method on this spectra dataset was 59% and average accuracy of 2 Ub/Ubls detection allowing mislocation was 95%. The proposed method showed the possibility of multi-Ub/Ubls identification with comprehensive search, classification, and integration of full mass differences. Because there is no previous method for multi-Ub/Ubls identification, this test couldn’t be compared with any previous method.Matching mass peaks and mass shift candidates from comprehensive analysis of full mass differences with theoretical mass shift series calculated with fragmentation patterns of Ub/Ubls boosts the sensitivity of detecting Ub/Ubls.Last, 2 PTMs including biological PTMs and Ub/Ubls are randomly attached to simulated spectra and the average accuracy of the proposed method was 52%. There is no previous method for identification of multiple PTMs including both non-peptide PTMs and peptide modifiers that generate complex fragmentation patterns. Thus, this test couldn’t be compared with any previous method.Although various PTMs are randomly attached on simulated spectra, the proposed method showed acceptable performance on identification of mixed PTMs including Ub/Ubls.

**Table 1:** Average accuracy of the proposed method on simulated spectra datasets generated with 4 different criteria.

	1 PTM	2 PTMs		
	Biological PTMs + Ub/Ubls	Biological PTMs	Ub/Ubls	Biological PTMs + Ub/Ubls

Average accuracy	91%	53%	59%	52%

### Identification of Ub/Ubls

For validation of ability of Ub/Ubls identification, mass spectra datasets from The Raught Lab Spectral Libraries that are built with SUMmOn are analyzed by the proposed method and successfully identified Ub, SUMO-1, SUMO-2/3, NEDD8, and diglycine modification from 36 mass spectra, which is exactly same with prior identification by T. Srikumar et al. [16]. The Raught Lab Spectral Libraries contains 478 consensus spectra with 36 mono-Ub/Ubls identified mass spectra: 4 Ub, 11 NEDD8, 3 SUMO-1, 11 SUMO-2/3 and 7 diglycine modifications. Mass spectra in The Raught Lab Spectral Libraries are CID spectra with high charge states up to 7+. The proposed method designed to cover charge states up to 10+ by calculating theoretical fragment ion mass with charge state variations and cluster matched fragment ions with mass shift classes. In the Spectral Libraries, precursor ion mass is existed on each mass spectrum which can be referred for accurate PTM identification. Mass shift paths that satisfy precursor ion mass are selected and PTMs in mass shift paths are identified by the proposed method. While T. Srikumar et al. used two PTM identification programs, X!Tandem for identification of diglycine modifications and SUMmOn for identification of Ub, NEDD8, SUMO-1, and SUMO-2/3, the proposed method successfully identified diglycine and Ub/Ubls without any other PTM identification program.

### Identification of PTMs

For validation of ability of PTM identification, mass spectra datasets that are used for QuickMod are analyzed by the proposed method. QuickMod searches PTMs using prebuilt library spectra, while the proposed method is based on theoretical peptide spectra match. Prebuilt library spectra can boost up identification accuracy but hard to cover mass spectra that are not existed in spectrum library. QuickMod provided test data sets that cover 5 PTMs (oxidation of methionine, phosphorylation of serine, threonine, or tyrosine, carbamidomethylation of cysteine, n-term acetylation and pyro-glu on n-term glutamic acid or glutamine) by extracting spectra from spectral libraries publicly available at NIST (http://peptide.nist.gov/) and ISB (http://www.peptideatlas.org[17]). Only one PTM is existed per spectrum in test data sets as QuickMod is not designed for identifying multiple PTMs. The proposed method analyzed doubly charged spectra (Mod_z2) from QuickMod test data sets with unrestricted search criteria. The accuracy of QuickMod with position agreement policy using theoretical peptide spectra match was 94%, while the accuracy of the proposed method with unrestricted search by theoretical peptide spectra match was 92.48%. The proposed method showed comparable performance of PTM identification with QuickMod, recently introduced PTM identification method.

### Detection of ubiquitination from tandem mass spectra datasets

**Figure 5:**
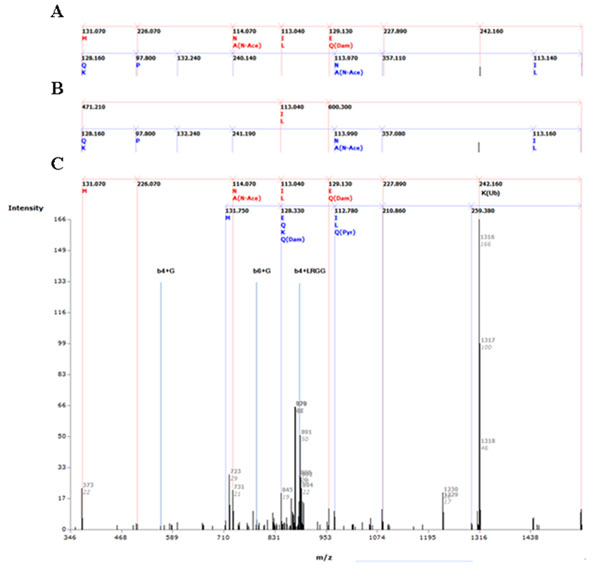
**Peak matching on Mass Spectrum ID 58395 from HUPO BPP Experiment #1735** (a) No PTM: BPP annotation (b) Ub on 15K: Analysis result by Mascot considering GlyGly (Ub) as a variable modification (c) Ub on 4K: Analysis result by the proposed method which is matched with Ubiprot annotation

To attempt identification of ubiquitination from HUPO BPP datasets, the list of ubiquitin substrates with ubiquitinated site were collected from E3miner [18] with minor tuning and Ubiprot [19]. Totally 38 ubiquitin substrates from human or mouse are collected with ubiqutination sites. Mass spectra matched with collected list of ubiquitin substrates in HUPO BPP dataset were analyzed using the proposed method. We found 6 ubiquitin substrates in HUPO BPP datasets: IPI00018352 (K4, K65, K71, and K157), IPI00291006 (K241), IPI00007188 (K146), IPI00011253 (K75, K90, K141, K197, and K202), and IPI00013296 (K78). From 82 datasets in HUPO BPP, 20 datasets (#1675, #1676, #1684, #1686, #1691, #1695, #1698, #1700, #1706, #1711, #1725, #1729, #1732, #1733, #1734, #1735, #1741, #1747, #1748, and #1749) contain 952 mass spectra from 6 ubiquitin substrates. Peptides including lysine residue in the middle of the peptide sequence that didn’t digested by trypsin were selected due to lysine modification such as Ub/Ubls or miscleavage. Trypsin is usually known to cleave next to lysine or arginine, but not before proline. However, recent study [20] reveals that trypsin may cleave lysine or arginine before proline. Therefore, we consider all cleavage sites including sites before proline. 144 spectra from 4 ubiquitin substrates (IPI00007188, IPI00011253, IPI00291006, and IPI00018352) are selected with the location of lysine residue. In the HUPO BPP datasets, both MS and MS/MS (tandem mass) spectra are existed and only tandem mass spectra are analyzed with the proposed method. Selected 118 tandem mass spectra are analyzed with the proposed method and found 12 spectra with ubiquitination. Ubiquitination of IPI00018352 on K4 and K71 are detected that are matched with collected ubiquitin substrates with ubiquitinated site [19,21]. For the comparison, Mascot MS/MS ions search is used for analysis of selected mass spectra and derived same result with the proposed method on most of selected mass spectra. For example, the proposed method and Mascot both identified Ub on K71 of IPI00018352 from Mass Spectrum ID 58402 from HUPO BPP Experiment #1735. However, different analysis results were derived from few mass spectra. Fig. 5 shows peak matching on Mass Spectrum ID 58395 from HUPO BPP Experiment #1735. There is no PTM information on BPP annotation for mass spectrum ID 58395. (Fig. 5.A) HUPO BPP datasets have considered only 5 kinds of chemical modifications with standard database search algorithms (SEQUEST, ProteinSolver, Mascot, and ProFound [22]) and there is no information for Ub. With considering diglycine modification as variable modification, Mascot identified Ub on K15. (Fig. 5.B) Though Mascot identified peptide correctly, only four y-ions are matched with spectrum from fourteen theoretical y-ions. However, our analysis result was Ub on K4, which is matched with information of collected ubiquitin substrates with ubiquitinated site. (Fig. 5.C) Eight b-ions, six y-ions, and three b-ions with fragmented Ub are matched with spectrum. *De novo* peptide sequencing based upon Ub on K4 generates longer sequence tags than Ub on K15.In total, by comparison of analysis results from Mascot and the proposed method, most of mass spectra that are identified with Ub by the proposed method are also identified with Ub by Mascot with high sequence coverage, though there were mislocations of Ub by Mascot in few mass spectra. In addition, mass spectra that are analyzed as no PTM by the proposed method are also identified with no PTM or mislocation of Ub with low sequence coverage by Mascot. Standard database search algorithms can identify peptides but hard to consider various PTMs altogether especially peptide modifiers. The proposed method showed possibility of detecting peptide modifiers from tandem mass spectra dataset generated by standard database search algorithms.

## Discussion

Identification of peptide modifiers such as ubiquitin and ubiquitin-like proteins requires additional techniques to handle various mass shifts made by fragments of peptide modifiers. The proposed method showed the ability of identification of multiple PTMs including peptide modifiers. The proposed method can be applied on analyzing tandem mass spectra datasets built with standard database search algorithms. Though precursor ion mass can help accurate identification of PTMs, the proposed method can try to identify PTMs when there are lack of information of precursor ion mass in the mass spectra. Applied to cluster computing, our method can analyze massive mass spectra datasets from high-throughput experiments. With development of mass spectrometer in terms of accuracy and sensitivity, overall performance of our method will be dramatically increased because smaller tolerance can effectively reduce computational artifacts of mass shift classes.

## Conclusions

Though identification of peptide modifiers becomes important to understand their roles in biological pathway regulations, identification of peptide modifiers with complex peak patterns from fragment ions of peptide modifiers remains a challenge. In this paper, we introduce a method for identification of peptide modifiers from tandem mass spectra with various PTMs with considering possible clues based on unrestricted PTM identification algorithm previously developed by authors and fragmented ion patterns of peptide modifiers. The proposed method is a novel method that can identify both PTMs that are not fragmented during fragmentation process and peptide modifiers that generate complex fragmentation patterns together from tandem mass spectra. Proposed method showed excellent performance in the test with simulated spectra having various PTMs including peptide modifiers and in the comparison with methods specialized for identification of PTMs or peptide modifiers. Not only identification of mono-Ub/Ubl or mono-PTM, identification of multiple PTMs including all known peptide modifiers can be applied with the proposed method.

## Competing interests

The authors declare that they have no competing interests.

## Authors' contributions

CK designed and implemented the proposed method and wrote the manuscript. GSY designed and directed this study, and reviewed the manuscript. All authors worked on and approved the final manuscript.

## References

[B1] Eng Jimmy K M A L Yates John R III An approach to correlate tandem mass spectral data of peptides with amino acid sequences in a protein database JASMS 1994 5 976 989 10.1016/1044-0305(94)80016-2 24226387

[B2] Yates JR Eng JK McCormack aL Mining genomes: correlating tandem mass spectra of modified and unmodified peptides to sequences in nucleotide databases Analytical chemistry 1995 67 18 3202 3210 10.1021/ac00114a016 8686885

[B3] Yates JR Eng JK McCormack aL Schieltz D Method to correlate tandem mass spectra of modified peptides to amino acid sequences in the protein database Analytical chemistry 1995 67 8 1426 1436 10.1021/ac00104a020 7741214

[B4] MacLean B Eng JK Beavis RC McIntosh M General framework for developing and evaluating database scoring algorithms using the TANDEM search engine Bioinformatics 2006 22 22 2830 2832 10.1093/bioinformatics/btl379 16877754

[B5] Ahrné E Müller M Lisacek F Unrestricted identification of modified proteins using MS/MS Proteomics 2010 10 4 671 686 10.1002/pmic.200900502 20029840

[B6] Hansen BT Davey SW Ham AJL Liebler DC P-Mod: an algorithm and software to map modifications to peptide sequences using tandem MS data Journal of proteome research 2005 4 2 358 368 10.1021/pr0498234 15822911

[B7] Chamrad DC Körting G Schäfer H Stephan C Thiele H Apweiler R Meyer HE Marcus K Blüggel M Gaining knowledge from previously unexplained spectra-application of the PTM-Explorer software to detect PTM in HUPO BPP MS/MS data Proteomics 2006 6 18 5048 5058 10.1002/pmic.200600189 16912973

[B8] Ahrné E Nikitin F Lisacek F Müller M QuickMod: a tool for open modification spectrum library searches Journal of proteome research 2011 10 7 2913 2921 10.1021/pr200152g 21500769

[B9] Pedrioli PGA Raught B Zhang X-d Rogers R Aitchison J Matunis M Aebersold R Automated identification of SUMOylation sites using mass spectrometry and SUMmOn pattern recognition software Nature Methods 2006 3 7 533 539 10.1038/nmeth891 16791211

[B10] Jeram SM Srikumar T Pedrioli PGa Raught B Using mass spectrometry to identify ubiquitin and ubiquitin-like protein conjugation sites Proteomics 2009 9 4 922 934 10.1002/pmic.200800666 19180541

[B11] Kang C Kim DJ Kim YR Yi GS Unrestricted identification of post translational modifications from tandem mass spectra datasets International Conference on Bioinformatics and Biomedical Technology 2010 244 247

[B12] Creasy DM Cottrell JS Unimod: protein modifications for mass spectrometry Proteomics 2004 4 6 1534 1536 10.1002/pmic.200300744 15174123

[B13] Witze ES Old WM Resing KA Ahn NG Mapping protein post-translational modifications with mass spectrometry Nature Methods 2007 4 798 806 10.1038/nmeth1100 17901869

[B14] Tsur D Tanner S Zandi E Bafna V Pevzner PA Identification of post-translational modifications by blind search of mass spectra Nature Biotechnology 2005 23 12 1562 1567 10.1038/nbt1168 16311586

[B15] Yan B Zout T Wang P Liu Z Emanuele VA II Olman V Xu Y A point-process model for rapid identification of post-translational modifications Pacific Symposium on Biocomputing 2006 11 327 338 17094250

[B16] Srikumar T Jeram SM Lam H Raught B A ubiquitin and ubiquitin-like protein spectral library Proteomics 2010 10 2 337 342 10.1002/pmic.200900627 19899083

[B17] Desiere F Deutsch EW King NL Nesvizhskii AI Mallick P Eng J Chen S Eddes J Loevenich SN Aebersold R The PeptideAtlas project Nucleic acids research 2006 34 Database issue D655 D658 16381952 10.1093/nar/gkj040 PMC1347403

[B18] Lee H Yi GS Park JC E3Miner: a text mining tool for ubiquitin-protein ligases Nucleic acids research 2008 36 Web Server issue W416 W422 18483079 10.1093/nar/gkn286 PMC2447767

[B19] Chernorudskiy AL Garcia A Eremin EV Shorina AS Kondratieva EV Gainullin MR UbiProt: a database of ubiquitylated proteins BMC bioinformatics 2007 8 126 10.1186/1471-2105-8-126 17442109 PMC1855352

[B20] Rodriguez J Gupta N Smith RD Pevzner PA Does trypsin cut before proline? Journal of proteome research 2008 7 1 300 305 10.1021/pr0705035 18067249

[B21] Meray RK Lansbury PT Reversible monoubiquitination regulates the Parkinson disease-associated ubiquitin hydrolase UCH-L1 The Journal of biological chemistry 2007 282 14 10567 10575 10.1074/jbc.M611153200 17259170

[B22] Zhang W Chait BT ProFound: an expert system for protein identification using mass spectrometric peptide mapping information Analytical chemistry 2000 72 11 2482 2489 10.1021/ac991363o 10857624

